# Patient-reported outcomes in head and neck cancer: a cross-sectional analysis of quality of life domains across early and advanced UICC stages

**DOI:** 10.1007/s00520-025-09204-3

**Published:** 2025-03-13

**Authors:** Moritz Allner, Atina Rak, Matthias Balk, Robin Rupp, Omar Almajali, Henriette Tamse, Juliane Gschossmann, Matti Sievert, Sarina Müller, Michael Koch, Heinrich Iro, Magdalena Gostian, Markus Hecht, Elisabeth Wimmer, Antoniu-Oreste Gostian

**Affiliations:** 1 Department of Otorhinolaryngology, Friedrich-Alexander-Universität (FAU), Head & Neck Surgery, Comprehensive Cancer Center Erlangen-EMN, Erlangen-Nuremberg, Germany; 2 Department of Anesthesiology and Intensive Care Medicine, Malteser Waldkrankenhaus St. Marien, Erlangen, Germany; 3 Department of Radiotherapy and Radiation Oncology, Saarland University Medical Center, Homburg/Saar, Germany; 4 Department of Otorhinolaryngology, Merciful Brothers Hospital St. Elisabeth, Head & Neck Surgery, 94315 Straubing, Germany; 5 Department of Otorhinolaryngology, Head and Neck Surgery, University of Erlangen-Nürnberg, Waldstraße 1, 91054 Erlangen, Germany

**Keywords:** Advanced-stage cancer, Disease stage, Early-stage cancer, Follow-up, Head and neck cancer, Individualized care, Patient-reported outcome, Physical symptoms, Psychological symptoms, Quality of life, Cancer, Survivorship

## Abstract

**Background:**

Head and neck cancer (HNC) patients experience a variety of post-treatment symptoms that affect their quality of life (QoL). This study aims to assess the most prevalent symptoms and their relationship to cancer stage (UICC I–IV) while identifying areas for targeted intervention.

**Methods:**

A cross-sectional study was conducted involving 340 HNC patients at the University Hospital Erlangen from January to December 2019. QoL and its domains were assessed using the German version of the University of Washington Quality of Life Questionnaire Version 4 (UW-QoL v.4), with comparisons made between early-stage (UICC I & II, *n* = 180) and advanced-stage (UICC III & IV, *n* = 160) patients. Statistical analysis examined differences in QoL and its individual domains.

**Results:**

Advanced-stage patients reported significantly greater impairments in several QoL domains, including swallowing (*p* = 0.003, *η*^2^ = 0.038), saliva production (*p* < 0.001, *η*^2^ = 0.104), and taste (*p* = 0.009, *η*^2^ = 0.030), compared to early-stage patients. Psychological symptoms, such as anxiety and mood disturbances, were prevalent across all stages, but no significant differences were found between early- and advanced-stage patients for pain, speech, mood, or anxiety (*p* > 0.05). Patient demographics, including age, gender, and comorbidities, were similar between groups. The greater impairments in QoL domains observed in advanced-stage patients are likely due to more intensive treatments, such as multimodal therapy and radiochemotherapy.

**Conclusion:**

Advanced-stage HNC patients experience a significantly higher burden of physical symptoms, particularly issues with swallowing, saliva, and taste, necessitating early and targeted interventions. Psychological issues are also prevalent and should be addressed in both early- and advanced-stage patients. Despite non-significant differences in some symptoms, their clinical relevance may still be important, particularly in individual cases. Comprehensive care, including physical and emotional support, is essential to improving long-term QoL for HNC patients. Further research should focus on longitudinal assessments and clinically meaningful thresholds for symptom management.

**Supplementary Information:**

The online version contains supplementary material available at 10.1007/s00520-025-09204-3.

## Introduction

Head and neck cancer (HNC) is the sixth most common malignancy worldwide [1]. Despite advancements in treatment, survival rates remain low, primarily due to initial late-stage diagnosis [2–4]. Recent developments in immunotherapy and technology offer new therapeutic possibilities and better outcomes. Concurrently, comprehensive support for HNC patients, taking medical and psychosocial care aspects into account, is increasingly being recognized as crucial [5]. However, HNC is often associated with significant impairments in Quality of Life (QoL), [6–12]. There is a substantial body of research on QoL and its domains in head and neck cancer (HNC) patients, particularly during the first years following diagnosis [13–18]. Numerous studies have investigated QoL within this timeframe, often highlighting significant physical and psychosocial impairments associated with cancer treatments. Long-term studies on HNC survivors reveal that persistent symptoms such as xerostomia, dysphagia, and speech difficulties continue to significantly affect their quality of life. These chronic issues remain prevalent even years after treatment, underscoring the lasting impact on survivors' overall well-being [19–33]. These differences underscore the importance of focusing on various stages of disease and treatment, including both early and advanced UICC stages, to better understand the full spectrum of patient outcomes. Moreover, the influence of UICC (Union internationale contre le cancer) staging, a global standard for classifying the extent of cancer spread, on QoL and its domains in cancer survivors remains under-evaluated.

For this reason, given the increasing incidence of HNC and the reported inadequacies in the care of this patient population—such as challenges in managing long-term impairments, fragmented psychosocial support, and gaps in access to symptom-oriented therapies—research in this area is of great importance to address the growing emphasis on symptom management and improvements in QoL [34].

By examining patients from a large HNC center with comprehensive care options—including medical, surgical, and psychosocial interventions—our aim is to better understand the care needs of HNC patients and inform strategies to address the unique requirements of this population.

The primary aim of our study was to evaluate the most prevalent symptoms in HNC patients after a variety of time periods following initial cancer treatment, specifically exploring the associations between UICC stage and QoL outcomes. Secondary objectives of our study included understanding the association between symptom prevalence and long-term QoL outcomes as well as identifying potential areas for intervention to improve the overall well-being of HNC patients, particularly in relation to cancer stage.

## Material and methods

This cross-sectional study received approval from the University of Erlangen’s Ethics Committee (No.: 486_18 B) and adhered to the Declaration of Helsinki as well as the STROBE guidelines [35, 36]. It was registered with the German Registry for Clinical Studies (DRKS) under application No.: 00017122.

From January 1st, 2019 to December 31st, 2019, a total of 503 patients attending follow-up appointments at the Department of Otolaryngology, Head & Neck Surgery at the University Hospital Erlangen were evaluated. After obtaining written informed consent, questionnaires were distributed to patients who had agreed to participate. Each patient was assessed at a single time point during their follow-up appointment. No predefined limit was set on the number of patients to be included, and all patients who met the inclusion criteria were invited to participate. Patients were included consecutively based on their arrival for consultation, with enrollment determined by their appointment schedules.

Patients were included if they were diagnosed with HNC of any UICC stage (I–IV) [37], were 18 years or older, had complete medical records, possessed adequate cognitive and language skills for completing the questionnaire independently, and provided written informed consent after receiving detailed study information. Exclusion criteria for this study were the following: below the legal age of consent, inability to complete the questionnaire independently, mental incapacity, and personal refusal to participate in the study. There was no specified time post-diagnosis for inclusion. Patients were included based on their attendance at follow-up appointments, regardless of the time elapsed since diagnosis. This allowed for a range of post-treatment experiences, from early recovery to long-term survivorship, offering a comprehensive view of QoL and its domains. This variability is important, as QoL fluctuates based on time since treatment, meaning individual recovery experiences and impairments in QoL domains may differ significantly.

The QoL of patients was assessed using the German version of the University of Washington Quality of Life Questionnaire (UW-QoL), Version 4 [38]. This disease-specific tool is designed to evaluate health-related QoL in HNC patients, encompassing 12 domains: pain, appearance, activity, recreation, swallowing, chewing, speech, shoulder function, taste, saliva, mood, and anxiety. Each domain is rated on a 0–100-point scale, where higher scores indicate better QoL. Additionally, the questionnaire includes three global questions on health-related and overall QoL, also scored on the same scale.

The UW-QoL allows for analysis via two subscales: physical and socioemotional function, which were used to distinguish between different types of QoL impairments [39]. In Tables 2 and 3, physical domains are highlighted in white, and socioemotional domains in gray. Both subscales were calculated and reported as mean, median, and 25th and 75th percentile values in Table 2.

The analysis compared QoL domains between early-stage (UICC I + II) and advanced-stage (UICC III + IV) patients. To provide a comprehensive understanding of impairments in QoL domains, we analyzed the data using both frequency distributions and summary statistics.

Supplementary Tables S1 and S2 present the frequency distributions for each QoL domain, showing the proportion of patients reporting no impairments (score = 100), moderate impairments, and severe impairments (score = 0). These distributions offer important insights into the prevalence and severity of impairments across specific domains, particularly for identifying subgroups of patients with extreme impairments that may be masked by average scores.

Table 2 focuses on summary statistics for QoL domains, presenting medians, 25th and 75th percentiles, means with standard deviations (SD), and statistical comparisons (p-values and effect sizes, η^2^) between early- and advanced-stage patients.

Table 3 summarizes composite scores for physical and socio-emotional subscales, as well as overall QoL and health-related QoL (HRQoL), comparing these scores between UICC stages. Statistical comparisons, including p-values and effect sizes, are reported for each subscale and domain. A difference of 7–10 points in QoL domain scores is generally considered clinically meaningful [39, 40].

By combining both frequency distributions and summary statistics, this approach provides a nuanced interpretation of QoL impairments: while mean scores reflect group-level trends, the frequency distributions in Supplementary Tables S1 and S2 highlight the clinical relevance of extreme scores, which are often most impactful for patient care.

Along with the questionnaire, clinical and oncological features, such as time from initial diagnosis to interview, regular medication intake, comorbidities, Eastern Cooperative Oncology Group (ECOG) status [41], cancer location, treatment method and -intention, and cancer recurrence, were surveyed from the patients' medical records. Comorbidities were categorized into cardiovascular, pulmonary, oncological, and neurological. Tumor staging was conducted using the eighth edition of the Tumor Node Metastasis (TNM) classification and the UICC classification [37]. Early tumor stages were defined as (UICC I and II) in contrast to the advanced stages (UICC III and IV). Treatment modalities were classified as follows: surgical treatment only, definitive chemoradiation, primary surgical treatment followed by adjuvant therapy (i.e., radiation or chemoradiation), and salvage surgery after the failure of any initial cancer treatment.

### Statistical computation

Statistical analyses were performed using IBM SPSS Statistics version 28.0 (IBM Corp., Armonk, NY, USA). A *p*-value < 0.05 was considered statistically significant.

Descriptive statistics were used to summarize continuous variables as means with standard deviations (SD) and categorical variables as frequencies and percentages. To compare differences between early-stage (UICC I & II) and advanced-stage (UICC III & IV) patients, chi-square tests were employed for categorical variables, with Cramér’s V used to assess effect sizes. For continuous variables, independent samples *t*-tests were conducted, and Cohen’s *d* was reported as the measure of effect size. This analysis allowed for a comprehensive comparison of patient characteristics across the two groups.

For the analysis of symptom severity and QoL, univariate analysis of variance (ANOVA)was used, with eta-squared (η^2^) serving as the effect size measure. η^2^ values help interpret the practical significance of the findings, where 0.01 indicates a small effect, 0.06 a moderate effect, and 0.14 or higher a large effect. Levene’s test assessed variance homogeneity, and any violations were noted with cautious interpretation. Additionally, a 10-point difference in QoL scores was considered clinically meaningful, following established guidelines [39, 40]. Significant results were highlighted with an asterisk (*) and bolded in tables.

## Results

### Patient collective

**Fig. 1:**
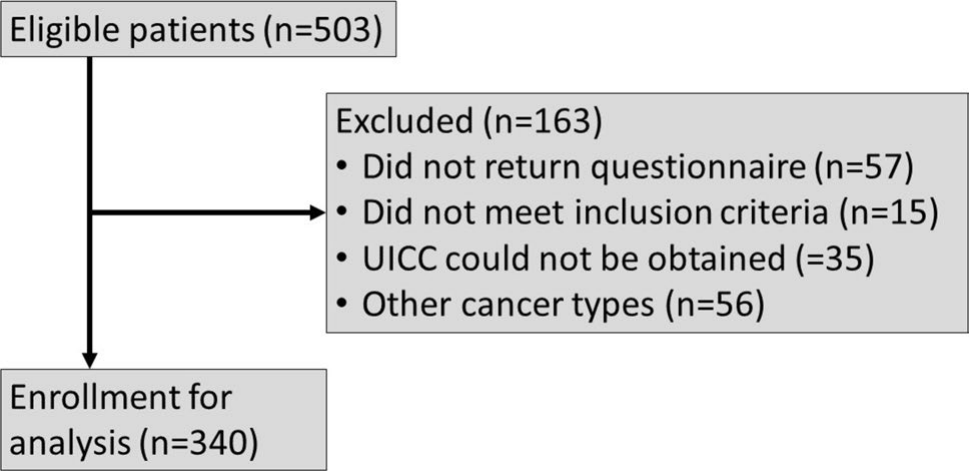
Study flow diagram

From 07/01/2019 to 12/31/2019, 503 patients attended their routine follow-up appointments at the Department of Otolaryngology, Head & Neck Surgery of the University Hospital Erlangen. Out of these, 340 patients were included in the final analysis. A total of 163 patients (32.4%) were excluded due to various reasons: 57 did not return the questionnaire, 15 did not meet the inclusion criteria, 35 had missing UICC staging information, and 56 had other cancer types (i.e., skin cancer, lymphoma) not relevant to this study (see Fig. 1).

### Patient characteristics (Table 1)

**Table 1:** Patient characteristics distributed according to UICC stages

*Variable*	UICC I & II n_total_ = 180	UICC III & IV n_total_ = 160	Statistical comparison
***Demography, n (%)***			
*Gender (female)*	39 (21.7%)	45 (28.1%)	*p* = 0.168, Cramér’s V = 0.075
*Mean age (years* ± *SD)*	61.9 ± 12.3	63.7 ± 11.7	*p* = 0.141, Cohen’s *d* = 0.117
*Time from initial diagnosis – interview (months, mean* ± *SD; min—max)*	42.7 ± 37.3 Min: 0, Max: 168	46.2 ± 46.2 Min: 0, Max: 248	*p* = 0.240, Cohen’s *d* = 0.084
***Medical history, n (%)***			
*Regular medication (Yes)*	109/162 (60.6%)	100/138 (62.5%)	*p* = 0.331, Cramér’s V = 0.056
*Cardiovascular (Yes)*	70/178 (39.3%)	61/157 (38.9%)	*p* = 0.930, Cramér’s V = 0.005
*Pulmonary (Yes)*	22/178 (12.4%)	24/157 (15.3%)	*p* = 0.437, Cramér’s V = 0.042
*Oncological (Yes)*	37/178 (20.8%)	42/157 (26.8%)	*p* = 0.199, Cramér’s V = 0.070
*Neurological (Yes)*	17/178 (9.6%)	22/157 (14.0%)	*p* = 0.204, Cramér’s V = 0.069
***ECOG functional status, n (%)***			
*ECOG 0*	126/157 (80.3%)	102/142 (71.8%)	*p* = 0.105, Cramér’s V = 0.123
*ECOG 1*	27/157 (17.2%)	30/142 (22.1%)	
*ECOG 2*	4/157 (2.5%)	10/142 (7.0%)	
***Cancer localization, n (%)***			
*Oral cavity*	17/180 (9.4%)	23/160 (14.4%)	***p*** = < 0.001*, Cramér’s V = 0.318
*Oropharynx*	64/190 (35.6%)	39/160 (24.4%)	
*Hypopharynx*	2/180 (1.1%)	24/160 (15.0%)	
*Larynx*	56/180 (31.1%)	32/160 (20.0%)	
*Sinonasal*	12/180 (6.7%)	13/160 (8.1%)	
*Nasopharynx*	1/180 (0.6%)	1/160 (0.6%)	
*Salivary glands*	24/180 (13.3%)	23/160 (14.4%)	
*Cancer of unknown primary*	2/180 (1.1%)	5/160 (3.1%)	
***Treatment modality, n (%)***			
*Surgery only*	92/180 (51.1%)	24/160 (15.0%)	***p*** ** < 0.001, Cramér’s V = 0.240**
*Definitive radiochemotherapy*	14/180 (7.8%)	48/160 (30.0%)	
*Surgery* + *aR(C)T*	72/180 (40.0%)	87/160 (54.4%)	
*Salvage surgery*	2/180 (1.1%)	1/160 (0.6%)	
***Treatment intention, n (%)***			
*Curative (Yes)*	180/180 (100%)	156/160 (97.5%)	***p*** = 0.033*, Cramér’s V = 0.115
***Recurrence of disease, n (%)***			
*Loco-regional recurrence*	16/178 (9.0%)	18/158 (11.4%)	*p* = 0.322, Cramér’s V = 0.058
*Distant recurrence*	2/178 (1.1%)	0/158 (0%)	

This table summarizes demographic, medical history, and treatment modality data for HNC patients, stratified by UICC stages I & II and III & IV. Abbreviations: Minimum (Min); Maximum (Max); Standard Deviation (SD); *P*-Value (*p*); Union internationale contre le cancer (UICC); Eastern Cooperative Oncology Group (ECOG); Radiochemotherapy (RCT); Adjuvant Radio(chemo)therapy (aR(C)T). Statistical comparisons are provided, including *p*-values and effect sizes (Cramér’s V for categorical variables and Cohen’s d for continuous variables). Significant values are highlighted in bold and marked with an asterisk (*).

Out of the 340 patients examined, 52.9% (*n* = 180) were diagnosed at early stages (UICC I and II), while 47.1% (*n* = 160) were diagnosed at advanced stages (UICC III and IV) of HNC. The early-stage group had 21.7% females (*n* = 39) compared to 28.1% females (*n* = 45) in the advanced-stage group, though this difference was not statistically significant (*p* = 0.168). The average age at the time of the interview was 61.9 ± 12.3 years for the early-stage group and 63.7 ± 11.7 years for the advanced-stage group, with no significant difference between the two (*p* = 0.141). The mean time from initial diagnosis to the interview was 42.7 ± 37.3 months (range: 0–168) for early-stage patients and 46.2 ± 46.2 months (range: 0–248) for advanced-stage patients, also showing no significant difference (*p* = 0.240).

There were no significant differences between the groups regarding comorbidities: cardiovascular (*p* = 0.930), pulmonary (*p* = 0.437), oncological (*p* = 0.199), and neurological conditions (*p* = 0.204). The distribution of ECOG functional status was similar between the groups (*p* = 0.105), with the majority of patients in both groups having an ECOG score of 0 (80.3% in the early-stage group and 71.8% in the advanced-stage group).

Regarding cancer localization, the most common sites were the oropharynx (35.6%) and the larynx (31.1%) in the early-stage group, and the oropharynx (24.4%) and larynx (20.0%) in the advanced-stage group. However, hypopharyngeal carcinomas were significantly more prevalent in the advanced-stage group (15.0%) compared to the early-stage group (1.1%) (*p* < 0.001).

Treatment modalities varied significantly by cancer stage (*p* < 0.001). Early-stage patients were more likely to undergo surgery only (51.1%) compared to advanced-stage patients (15.0%). Conversely, advanced-stage patients more frequently received definitive radiochemotherapy (early-stage: 7.8% vs. advanced-stage: 30.0%) or multimodal therapy (surgery plus adjuvant radio(chemo)therapy) (early-stage: 40.0% vs. advanced-stage: 54.4%). Salvage surgery was rare and similar between groups (early-stage: 1.1% vs. advanced-stage: 0.6%).

The treatment intention was curative in 100% of the early-stage patients and 97.5% of the advanced-stage patients, with a statistically significant difference (*p* = 0.033). The rates of loco-regional recurrence were 9.0% in the early-stage group and 11.4% in the advanced-stage group (*p* = 0.322), while distant recurrence occurred in 1.1% of early-stage patients and none in advanced-stage patients.

### Frequency of self-reported symptoms

In early-stage HNC patients, most reported minimal impairments in QoL domains, with the highest scores (indicating no issues) seen in chewing (80.7%), shoulder function (77.8%), and swallowing (64.9%). However, greater impairments were noted in taste (3.0% reporting complete loss, 7.2% moderate impairment) and saliva production (1.9% reporting no saliva, 10.7% significant reductions). Psychological issues, such as mood disturbances (11.2%) and anxiety (8.1%), were less frequent but present. Tables S1 & S2

In contrast, advanced-stage patients reported significantly greater impairments across domains. While chewing (70.7%), shoulder function (68.9%), and speech (52.7%) remained relatively preserved, taste and saliva were notably affected. Complete taste loss was reported by 5.6%, and severe reductions in saliva were reported by 28.7%, with 5.6% experiencing a total lack of saliva.

Psychological issues were also more prominent in advanced-stage patients, with anxiety reported by 48.7% and mood disturbances by 17.9%, reflecting a heightened burden in both physical and psychological domains.

### Domain-specific quality of life comparison between early and advanced stage HNC patients

Symptom severity comparisons between early-stage (UICC I + II, *n* = 180) and advanced-stage (UICC III + IV, *n* = 160) HNC patients revealed several statistically significant differences across key domains. Notably, pain, speech, mood, and anxiety showed no significant differences between the groups (*p* > 0.05). Table 2

**Table 2:** Symptom-specific quality of life comparison between early and advanced stage HNC patients

**Domain**	***UICC I* + *II (n***_total_** = *180)***		***UICC III* + *IV (n***_total_** = *160)***		**Statistical comparison**
	Md [25.;75.P.]	Mean ± SD	Md [25.;75.P.]	Mean ± SD	ANOVA
*Pain*	100 (75;100)	83.9 ± 21.7	75 (50;100)	78.8 ± 23.8	*p* = 0.241, *η*^2^ = 0.006
***Appearance***	100 (75;100)	86.2 ± 19.1	75 (75;100)	78.5 ± 20.4	*p* = 0.003*, *η*^**2**^**= 0.038**
***Activity***	75 (50;100)	78.3 ± 23.0	75 (50;100)	68.7 ± 24.8	*p* = 0.005*, *η*^**2**^** = 0.034**
***Recreation***	100 (75;100)	82.1 ± 23.1	75 (50;100)	72.7 ± 26.6	*p* = 0.004*, *η*^**2**^** = 0.037**
***Swallowing***	100 (75;100)	88.7 ± 17.7	75 (75;100)	80.8 ± 18.9	*p* = 0.003*, *η*^**2**^** = 0.038**
***Chewing***	100 (100;100)	89.4 ± 22.5	100 (50;100)	83.2 ± 27.8	*p* = 0.043*, *η*^**2**^** = 0.018**
*Speech*	100 (70;100)	85.3 ± 22.2	100 (70;100)	82.3 ± 21.9	*p* = 0.874, *η*^2^= 0.000
***Shoulder***	100 (100;100)	89.1 ± 23.6	100 (70;100)	81.1 ± 31.5	*p* = 0.011*, *η*^**2**^**= 0.029**
***Taste***	100 (70;100)	82.5 ± 25.2	70 (70;100)	72.5 ± 29.6	*p* = 0.009*, *η*^**2**^** = 0.030**
***Saliva***	100 (70;100)	82.7 ± 25.5	70 (30;100)	64.4 ± 31.7	*p* < 0.001*, *η*^**2**^** = 0.104**
*Mood*	75 (75;100)	75.6 ± 24.2	75 (50;100)	73.1 ± 22.6	*p* = 0.401, *η*^2^ = 0.003
*Anxiety*	70 (70;100)	79.0 ± 22.3	70 (70;100)	74.2 ± 26.0	*p* = 0.106, *η*^2^ = 0.012

Comparison of Symptom-specific QoL domains between early (UICC I + II) and advanced (UICC III + IV) stage HNC patients using ANOVA, with data collected via the German version of the University of Washington Quality of Life (UW-QoL) questionnaire Version 4. The results are reported as Median (Md), Interquartile Range [25th; 75th Percentile], Mean (M) ± Standard Deviation (SD), *P*-Value (*p*), and Effect Size (*η*^2^ or eta-squared). Statistically significant values are highlighted in bold and marked with an asterisk (*).

Levene’s test showed violations of the homogeneity of variances assumption for Chewing (*p* < 0.001), Shoulder (*p* < 0.001), and Saliva (*p* = 0.007). Interpretations of these results should be made with caution due to heteroscedasticity.

Symptoms are color-coded to differentiate between physical function (white) and social-emotional function (gray) [39]

Among the significant findings, the saliva domain demonstrated the most pronounced and clinically relevant difference (*p* < 0.001, *η*^2^ = 0.104), with early-stage patients reporting significantly less severe issues (Md = 100, IQR 70–100, M ± SD = 82.7 ± 25.5) compared to advanced-stage patients (Md = 70, IQR 30–100, M ± SD = 64.4 ± 31.7). The clinically meaningful difference of more than 18 points underscores the significant impact of advanced-stage disease on saliva production and QoL.

The appearance domain also showed a substantial difference (*p* = 0.003, *η*^2^ = 0.038), with early-stage patients reporting better scores (Md = 100, IQR 75–100, M ± SD = 86.2 ± 19.1) than advanced-stage patients (Md = 75, IQR 75–100, M ± SD = 78.5 ± 20.4). This difference is clinically meaningful, reflecting the higher physical and emotional burden experienced by advanced-stage patients related to appearance.

Further significant differences were noted in swallowing (*p* = 0.003, *η*^2^ = 0.038), with early-stage patients again reporting fewer issues (Md = 100, IQR 75–100, M ± SD = 88.7 ± 17.7) than advanced-stage patients (Md = 75, IQR 75–100, M ± SD = 80.8 ± 18.9). This difference also reached a clinically meaningful threshold, highlighting the increased swallowing difficulties in advanced-stage patients, likely due to more intensive treatments.

Other domains with significant differences included activity (*p* = 0.005, *η*^2^ = 0.034), recreation (*p* = 0.004, *η*^2^ = 0.037), chewing (*p* = 0.043, *η*^2^ = 0.018), shoulder function (*p* = 0.011, *η*^2^ = 0.029), and taste (*p* = 0.009, *η*^2^ = 0.030), where early-stage patients generally reported less severe symptoms. These differences, although statistically significant, had smaller effect sizes compared to saliva, swallowing, and appearance, yet still reflect the greater impairments in QoL domains faced by advanced-stage patients..

In contrast, for symptoms such as pain, speech, mood, and anxiety, despite differences observed, these did not reach statistical significance, and the clinical relevance of these differences remains uncertain.

### Quality of life

Patients with early-stage disease reported significantly better physical scores (Md = 90.8, IQR 76.6–100, M ± SD = 86.6 ± 14.1) than those with advanced-stage disease (Md = 79.1, IQR 68.3–90.8, M ± SD = 78.6 ± 14.6), with a statistically significant difference (*p* < 0.001, *η*^2^ = 0.076). Similarly, early-stage patients fared better in terms of socioemotional well-being (Md = 84.1, IQR 74.1–95.8, M ± SD = 81.9 ± 16.1) compared to advanced-stage patients (Md = 79.1, IQR 61.6–90.8, M ± SD = 75.7 ± 17.6). The difference was statistically significant (*p* = 0.003, *η*^2^ = 0.042), though the effect size was smaller than in the physical domain.

When comparing health-related QoL to pre-cancer status, no statistically significant difference was observed between early- and advanced-stage patients (*p* = 0.162, *η*^2^ = 0.009), with both groups showing similar medians (early-stage: Md = 50, IQR 25–100, M ± SD = 53.9 ± 33.8; advanced-stage: Md = 50, IQR 25–75, M ± SD = 47.2 ± 35.7).

Both groups reported similar health-related quality of life (HRQoL) over the past 7 days, with no significant difference between early-stage and advanced-stage patients (*p* = 0.168, *η*^2^ = 0.009). Early-stage patients had a median HRQoL score of 60 (IQR 40–80, M ± SD = 56.9 ± 22.9), while advanced-stage patients reported a lower median score of 40 (IQR 40–60, M ± SD = 52.1 ± 20.3), though this difference was not statistically significant.

Both groups reported similar overall QoL scores in the past 7 days, with no significant difference (*p* = 0.089, *η*^2^ = 0.014). Early-stage patients had a median score of 60 (IQR 40–80, M ± SD = 61.4 ± 22.2), while advanced-stage patients reported a median of 60 as well (IQR 40–80, M ± SD = 56.9 ± 21.8). Table 3

**Table 3:** Physical, socioemotional, health-related and overall quality of life scores in HNC patients

	***UICC I* + *II (n***_total_** = *120)***		***UICC III* + *IV (n***_total_** = *92)***		**Statistical comparison**
	**Md [25.;75.P.]**	**Mean ± SD**	**Md [25.;75.P.]**	**Mean ± SD**	ANOVA
***Physical complex***	90.8 (76.6; 100)	86.6 ± 14.1	79.1 (68.3; 90.8)	78.6 ± 14.6	*p* < 0.001*; *η*^**2**^** = 0.076**
***Socioemotional complex***	84.1 (74.1; 95.8)	81.9 ± 16.1	79.1 (61.6; 90.8)	75.7 ± 17.6	*p* = 0.003*; *η*^**2**^** = 0.042**
*HRQoL compared to pre-cancer*	50 (25; 100)	53.9 ± 33.8	50 (25; 75)	47.2 ± 35.7	*p* = 0.162; *η*^2^ = 0.009
*HRQoL over the past 7 days*	60 (40; 80)	56.9 ± 22.9	40 (40; 60)	52.1 ± 20.3	*p* = 0.168; *η*^2^ = 0.009
*Overall QoL over the past 7 days*	60 (40; 80)	61.4 ± 22.2	60 (40; 80)	56.9 ± 21.8	*p* = 0.089; *η*^2^ = 0.014

## Discussion

The primary objective of this study was to assess the most prevalent post-treatment symptoms in HNC patients and their relationship with UICC stage. Advanced-stage patients consistently reported more severe physical symptoms, particularly difficulties with swallowing, saliva production, and taste, indicating a greater overall burden compared to early-stage patients. Importantly, there were no significant differences in demographics, such as age, gender, or comorbidities, between early- and advanced-stage patients. This suggests that the observed differences in QoL are more likely due to disease stage and treatment regimens rather than patient characteristics, reinforcing the impact of cancer progression and therapy intensity on QoL. More intensive treatments, such as multimodal therapy and higher-dose radiochemotherapy, likely contribute to greater impairments, particularly in swallowing, saliva production, and taste.

Secondary objectives included evaluating how the prevalence of impairments in QoL domains impacts long-term QoL and identifying areas for intervention to alleviate these impairments. Targeted interventions should prioritize the most severe symptoms in advanced-stage patients, such as dysphagia, xerostomia, and taste impairment. Addressing these specific symptoms earlier in treatment could potentially mitigate long-term QoL decline. Psychological support is also critical, as mood disturbances and anxiety were prevalent across both early- and advanced-stage patients, emphasizing the need for comprehensive care that addresses both physical and emotional well-being.

While significant differences were observed in most domains, symptoms such as pain, speech, mood, and anxiety showed no significant variation between early and advanced-stage patients. Although they did not show statistically significant differences, their clinical relevance should not be overlooked. For instance, in both groups, nearly half of the patients reported moderate to severe pain, which warrants clinical attention despite not being significant.

While the mean difference in pain scores fell below this threshold, individual patients with severe symptoms should still receive targeted interventions. Similarly, even though anxiety and mood disturbances showed no significant variation (*p* > 0.05), the prevalence of these issues across stages suggests the need for psychological support in clinical practice.

Despite pain being reported by nearly half of the patients, no significant difference was observed between early and advanced stages. However, pain is widely recognized as a significant detriment to QoL, highlighting the need for ongoing pain management strategies [42–44].

For appearance, our findings align with Gamba et al. [45], who reported significant self-image changes in 45% of HNC patients post-surgery. While our study also found appearance-related concerns in both early- and advanced-stage patients, the prevalence differed, with only 36.4% of advanced-stage and 58.6% of early-stage patients reporting no such concerns.

Our study is consistent with Chaturvedi et al. [46], who also found that advanced-stage HNC patients frequently experienced activity limitations, which can lead to social isolation. This underscores the social impact of HNC, emphasizing the need for long-term supportive interventions.

Recreation scores were significantly worse in advanced-stage HNC patients. Previous studies have shown that difficulties with eating, speech, and fatigue contribute to these limitations, as patients find it harder to engage in social and recreational activities post-treatment [19]. Vartanian et al. noted that these deficits persist long-term, particularly in those undergoing aggressive treatments, further reducing patients’ overall well-being [47].

Swallowing difficulties were significantly more common in advanced-stage patients, reflecting known risk factors such as tumor localization and the use of adjuvant radiotherapy [48–51]. Additionally, radiation therapy is associated with deteriorating dental health, often affecting chewing function, as well as increasing the likelihood of trismus, particularly after multimodal treatments [52, 53]. In line with other studies, advanced-stage patients in our cohort also reported higher rates of xerostomia [54, 55], which has been strongly linked to higher radiation doses.

Taste impairment was another domain where advanced-stage patients reported significantly worse outcomes. As documented in Baharvand et al. [56] and Alvarez-Camacho et al. [57], taste alterations can severely affect nutrition, eating behavior, and overall psychological well-being, necessitating early and effective management.

Shoulder issues, though infrequent in both patient cohorts, are significant due to potential accessory nerve damage during treatment, particularly neck dissection. This can impair mobility and reduce QoL. Studies by Nibu et al. and Bradley et al. [8, 58] confirm the long-term impact of shoulder morbidity, emphasizing the need for early rehabilitation to mitigate these effects and improve outcomes [8, 43, 58].

Both anxiety and mood disturbances were prevalent across stages, with advanced-stage patients slightly more affected. This finding suggests that psychological care should be integral to HNC management, regardless of cancer stage. Tailored interventions focusing on emotional recovery could improve QoL outcomes across all stages of the disease [59–62].

The results regarding QoL demonstrate significant reductions in both physical and socioemotional QoL for patients with advanced-stage HNC (UICC III/IV), as shown in Table 3. Advanced-stage patients exhibited notably lower scores in the physical domain compared to early-stage patients, consistent with findings from previous studies, which highlight the greater physical impairments in advanced cancer [38].

Similarly, socioemotional QoL was significantly lower in UICC III/IV patients, corroborating research by Rogers et al. and Sayed et al. on the emotional and social burdens of advanced disease. While health-related and overall QoL in the last 7 days did not show significant differences, the overall trend underscores the profound impact of cancer progression on both physical functioning and emotional well-being [38, 63].

This study has several limitations. The cross-sectional design restricts causal inferences, as data were collected at a single time point, preventing us from observing changes in QoL impairments over time. Additionally, the potential for survivorship bias exists, as patients with more advanced or severe disease, who may not have survived or were too ill to attend follow-ups, are likely underrepresented. The reliance on self-reported data introduces recall bias, as patients may misreport or understate their symptoms, especially with long time gaps since treatment. Moreover, selection bias is a concern since only those attending regular outpatient check-ups were included, likely excluding patients with recurrent, advanced disease or severe comorbidities.

In addition, Levene’s test indicated violations of the assumption of homogeneity of variances for Chewing (*p* < 0.001), Shoulder (*p* < 0.001), and Saliva (*p* = 0.007), suggesting the presence of heteroscedasticity. This limitation may affect the reliability of the statistical analyses, particularly for these variables, and interpretations of results related to them should be made with caution. Heteroscedasticity may increase the likelihood of Type I or Type II errors, potentially skewing effect size estimations. Future research should consider using statistical methods that are robust to these violations, such as generalized linear models or bootstrapping, to ensure more accurate interpretations.

Despite these limitations, the large and diverse cohort provides valuable insights into QoL and its domains across different UICC stages of HNC. Future research should adopt longitudinal approaches and focus on clinical relevance while addressing these biases.

## Conclusion

This study highlights the greater impairments in QoL domains among advanced-stage HNC patients, particularly in swallowing, saliva production, and taste, likely due to more intensive treatments. Early intervention targeting these impairments is essential to improving long-term QoL. Psychological issues like mood disturbances and anxiety were prevalent across all stages, underscoring the need for comprehensive care addressing both physical and emotional well-being. Even symptoms that did not show statistically significant differences, such as pain and speech, should not be disregarded, as they may still have clinical significance for individual patients. Clinicians must focus on treating all relevant symptoms to provide holistic, patient-centered care. While this study is limited by its cross-sectional design and potential biases, it offers valuable insights into the QoL challenges faced by HNC patients. Future research should adopt longitudinal approaches and consider clinically meaningful thresholds to better inform patient care.

## Supplementary Information

Below is the link to the electronic supplementary material.

Supplementary file1 (DOCX 32 KB)

## Data Availability

The datasets used and/or analyzed during the current study are available from the corresponding author on reasonable request, because the data cannot be anonymized.

## Abbreviations

CI: 95% Confidence interval
aRCT: Adjuvant radio(chemo)therapy
CUP: Cancer of unknown primary
Prct: Definitive radiochemotherapy
ECOG: Eastern Cooperative Oncology Group
HNC: head and neck cancer
I: Intensity
IQR: Interquartile range
M: Mean
Md: Median
TNM: Tumor Node Metastasis classification
SD: Standard deviation
UICC: Union Internationale Contre le Cancer
UW-QoL: University of Washington-Quality of Life

## Declarations

This article was created using the STROBE guidelines.
