# Supplementary material for: Patient-reported outcomes in head and neck cancer: a cross-sectional analysis of quality of life domains across early and advanced UICC stages
Source: Support Care Cancer. 2025 Mar 13;33(4):278. doi: 10.1007/s00520-025-09204-3 (PMC11906576; doi:10.1007/s00520-025-09204-3)
Supplement: Supplementary file 1 — Supplementary file1 (DOCX 32 KB) [file 520_2025_9204_MOESM1_ESM.docx]

| ***Table S1. Frequency of self-reported symptoms in patients with early-stage disease, n (%)*** | | | | | | | |
| --- | --- | --- | --- | --- | --- | --- | --- |
| Domain | 0 | 25 | 30 | 50 | 70 | 75 | 100 |
| Pain, n = 176/180 | 2 (1.1) | 4 (2.3) |  | 22 (12.5) |  | 49 (27.8) | 99 (56.3) |
| Appearance, n = 174/180 | 0 (0) | 5 (2.9) |  | 14 (8.0) |  | 53 (30.5) | 102 (58.6) |
| Activity, n = 173/180 | 0 (0) | 7 (4.0) |  | 42 (24.3) |  | 45 (26.0) | 79 (45.7) |
| Recreation, n = 172/180 | 0 (0) | 14 (8.1) |  | 14 (8.1) |  | 53 (30.8) | 91 (52.9) |
| Swallowing, n = 174/180 | 5 (2.9) |  | 7 (4.0) |  | 49 (28.2) |  | 113 (64.9) |
| Chewing, n = 166/180 | 3 (1.8) |  |  | 29 (17.5) |  |  | 134 (80.7) |
| Speech, n = 158/180 | 2 (1.3) |  | 10 (6.3) |  | 47 (29.7) |  | 99 (62.7) |
| Shoulder, n = 153/180 | 4 (2.6) |  | 9 (5.9) |  | 21 (13.7) |  | 119 (77.8) |
| Taste, n = 166/180 | 5 (3.0) |  | 12 (7.2) |  | 52 (31.3) |  | 97 (58.4) |
| Saliva, n = 159/180 | 3 (1.9) |  | 17 (10.7) |  | 42 (26.4) |  | 97 (61.0) |
| Mood, n = 161/180 | 1 (0.6) | 17 (10.6) |  | 16 (9.9) |  | 70 (43.5) | 57 (35.4) |
| Anxiety n = 172/180 | 2 (1.2) |  | 14 (8.1) |  | 81 (47.1) |  | 75 (43.6) |

**Table S1.** Frequency of self-reported symptoms in patients with early-stage disease (UICC I + II, n = 180), based on the German version of the University of Washington Quality of Life Questionnaire Version 4 (UW-QoL v.4), presented as number and percentage (%).

This table presents the distribution of symptom severity in early-stage HNC patients, across multiple domains, categorized into six severity levels: 0, 25, 30, 50, 70, 75, and 100. The frequency of reported symptoms is shown as the number of patients (n) and percentages (%) for each domain.

Symptoms are color-coded to differentiate between physical function (white) and social-emotional function (gray) [39]. Blacked-out spaces indicate that the respective option is not applicable for that particular scale.

| ***Table S2. Frequency of self-reported symptoms in patients with advanced-stage disease, n (%)*** | | | | | | | |
| --- | --- | --- | --- | --- | --- | --- | --- |
| Symptom | 0 | 25 | 30 | 50 | 70 | 75 | 100 |
| Pain, n = 155/160 | 1 (0.6) | 8 (5.2) |  | 30 (19.4) |  | 43 (27,7) | 73 (47.1) |
| Appearance, n = 154/160 | 1 (0.6) | 5 (3.2) |  | 21 (13.6) |  | 71 (46.1) | 56 (36.4) |
| Activity, n = 152/160 | 3 (2.0) | 9 (5.9) |  | 49 (32.2) |  | 47 (30.9) | 44 (28.9) |
| Recreation, n = 153/160 | 2 (1.3) | 20 (13.1) |  | 22 (14.4) |  | 55 (35.9) | 54 (35.3) |
| Swallowing, n = 149/160 | 7 (4.7) |  | 8 (5.4) |  | 77 (51.7) |  | 57 (38.3) |
| Chewing, n = 140/160 | 6 (4.3) |  |  | 35 (25.0) |  |  | 99 (70.7) |
| Speech, n = 146/160 | 1 (0.7) |  | 11 (7.5) |  | 57 (39.0) |  | 77 (52.7) |
| Shoulder, n = 135/160 | 7 (5.2) |  | 20 (14.8) |  | 15 (11.1) |  | 93 (68.9) |
| Taste, n = 143/160 | 8 (5.6) |  | 21 (14.7) |  | 55 (38.5) |  | 59 (41.3) |
| Saliva, n = 143/160 | 8 (5.6) |  | 41 (28.7) |  | 47 (32.9) |  | 47 (32.9) |
| Mood, n = 145/160 | 0 (0) | 13 (9.0) |  | 26 (17.9) |  | 65 (44.8) | 41 (28.3) |
| Anxiety, n = 154/160 | 6 (3.9) |  | 16 (10.4) |  | 75 (48.7) |  | 57 (37.0) |

**Table S2.** Frequency of self-reported symptoms in patients with advanced-stage disease (UICC I + II, n = 160), based on the German version of the University of Washington Quality of Life Questionnaire Version 4 (UW-QoL v.4), presented as number and percentage (%).

This table presents the distribution of symptom severity in early-stage HNC patients, across multiple domains, categorized into six severity levels: 0, 25, 30, 50, 70, 75, and 100. The frequency of reported symptoms is shown as the number of patients (n) and percentages (%) for each domain.

Symptoms are color-coded to differentiate between physical function (white) and social-emotional function (gray) [39]. Blacked-out spaces indicate that the respective option is not applicable for that particular scale.
